# *In vivo* Real-Time Mass Spectrometry for Guided Surgery Application

**DOI:** 10.1038/srep25919

**Published:** 2016-05-18

**Authors:** Benoit Fatou, Philippe Saudemont, Eric Leblanc, Denis Vinatier, Violette Mesdag, Maxence Wisztorski, Cristian Focsa, Michel Salzet, Michael Ziskind, Isabelle Fournier

**Affiliations:** 1 Univ. Lille, INSERM, U1192 - Laboratoire Protéomique, Réponse Inflammatoire et Spectrométrie de Masse-PRISM, F-59000 Lille, France; 2 Univ. Lille, CNRS, UMR 8523 - PhLAM - Physique des Lasers Atomes et Molécules, F-59000 Lille, France; 3 Department of Gynecology Oncology, Cancer Center Oscar Lambret, Lille, France; 4 Département Universitaire de Gynécologie Obstétrique, Service de chirurgie gynécologique, Hôpital Jeanne de Flandre, CHRU de Lille, 59037 Lille Cedex, France

## Abstract

Here we describe a new instrument (SpiderMass) designed for *in vivo* and real-time analysis. In this instrument ion production is performed remotely from the MS instrument and the generated ions are transported in real-time to the MS analyzer. Ion production is promoted by Resonant Infrared Laser Ablation (RIR-LA) based on the highly effective excitation of O-H bonds in water molecules naturally present in most biological samples. The retrieved molecular patterns are specific to the cell phenotypes and benign versus cancer regions of patient biopsies can be easily differentiated. We also demonstrate by analysis of human skin that SpiderMass can be used under *in vivo* conditions with minimal damage and pain. Furthermore SpiderMass can also be used for real-time drug metabolism and pharmacokinetic (DMPK) analysis or food safety topics. SpiderMass is thus the first MS based system designed for *in vivo* real-time analysis under minimally invasive conditions.

Mass Spectrometry (MS) is one of the analytical techniques with the widest accessible range of applications. Its strength relies in its ability to retrieve molecular information from complex mixtures with the possibility to identify each detected compound, as well as the broad panel of MS-based techniques that allow the design of instruments of various geometries, performances, characteristics and capabilities. MS can be used for applications as remote as analyzing solar wind during space missions^1^,^2^ or trying to get a deeper coverage of the proteome of human cells^3^,^4^. The last decades have witnessed the important changes within the field of MS slowly moving from conventional analysis of extracted samples introduced into the mass spectrometer to the analysis of sample tissue pieces. This has led to the emergence and development of various imaging technologies starting with SIMS MS imaging in the 60’s^5^,^6^ to direct MALDI MS analyses^7^ and MALDI MS imaging^8^. More recently, because imaging techniques are often performed under high vacuum conditions that require a careful preparation of the samples before analysis, many novel ion production methods operating under atmospheric pressure conditions such as AP-MALDI^9^,^10^, Desorption ElectroSpray Ionization (DESI)^11^ or Laser Ablation ElectroSpray Ionization (LAESI)^12^ were introduced. The growing number of studies using surface analysis demonstrate the importance of retrieving molecular information and correlate to a location on the surface (spatially resolved analysis). In biological and clinical applications, it has been clearly demonstrated that the spatially resolved molecular information retrieved can be correlated to a physiological or physiopathological event^13^,^14^,^15^,^16^,^17^. Even though enormous progress has been achieved with the possibility to analyze surfaces or sometimes small pieces of material extracted from bigger objects or living organisms, the next challenge for MS is to translate from *ex vivo* analysis to *in vivo* conditions at the level of human beings. Being able to get molecular information directly *in vivo* is an important goal and already exponentially broaden the possibilities of MS. DESI was one of the first technologies used to apply MS in examining living organisms^18^. DESI can be applied to search for pesticides from intact fruits or vegetables or by using proper solvents for finding traces of drugs or gunpowder directly from the hands^18^,^19^,^20^. Many other technologies have emerged in the past decade^21^, some of them have found applications in daily life such as PaperSpray combined with a miniaturized MS instrument that is implemented at airport security control gates^22^,^23^. The next Holy Grail to be reached for MS is then to be able to perform *in vivo* analysis of human beings for healthcare considerations and in particular for diagnostic purposes. The difficulty of the challenge is to make MS compatible with *in vivo* analysis in the human body. For example, if conventional DESI can be used for screening small molecules from the skin surface, it cannot properly be used for surgery purposes because of the operating conditions and the geometry of the ion source. Over the past five years few different solutions were proposed to facilitate the transition of MS analysis towards *in vivo* conditions for clinical applications in the surgery room. The first of these is the iKnife (Intelligent Knife) system^24^ that is based on Rapid Evaporative Ionization Mass Spectrometry (REIMS)^25^. In this system the smoke produced by cutting tissues with an electric scalpel^25^ or electric forceps^26^ is collected by aspiration and transmitted to a QTOF MS instrument. REIMS allows for the real-time direct monitoring *in vivo* from patient tissues through intact as well as fragments of lipids molecular profiles. By developing a database and appropriate bioinformatics solution the ability of REIMS for diagnostic purpose during surgery was demonstrated^27^. More recently, a modified DESI source for translation to *in vivo* surgery applications was described^28^. *In vivo* DESI uses a pure water spray and no electric field but high flow rate. Although the mechanism leading to charged particle formation has not really been elucidated for *in vivo* DESI, similar molecular patterns of lipids under *ex vivo* conditions could be obtained. It is to be noted that *in vivo* DESI has not yet been proven *in vivo* on the human body although the system size is compatible with endoscopic conditions. Another technique recently introduced is remote LAESI^29^, where the surface can be remotely analyzed from the instrument. Because the ablated material must be transferred to be captured by the charged droplet spray of the ESI source, this technique necessitates that laser ablation be performed inside an enclosure. As such, its *in vivo* application is restricted to samples that can be placed inside an enclosure, as tree leaves or flowers. Among these techniques, only the REIMS technology has been proven for use in the human body, although REIMS remains relatively invasive since it is connected to a surgical instrument such as an electric scalpel.

Here we developed for the first time an instrument allowing real-time analysis under *in vivo* conditions with minimal damage based on laser ablation and mass spectrometry and with the ability to provide molecular characteristic data on samples *in-vivo* ranging from apple skin analysis for food safety and human skin analysis for real-time Drug Metabolism & Pharmacokinetics (DMPK) or cosmetic applications to considered *e.g*. human patient tissues for clinical applications in the field of guided surgery. In other words SpiderMass is a MS-based system for *in vivo* real-time analysis with large scale applications. One of the major is that this system based on laser ablation with remote ion formation and real-time retro-transportation of the gas phase ions fulfills the requirements for applications to human body for surgery purposes.

## Results

### SpiderMass instrument for real-time MS

**Figure 1:**
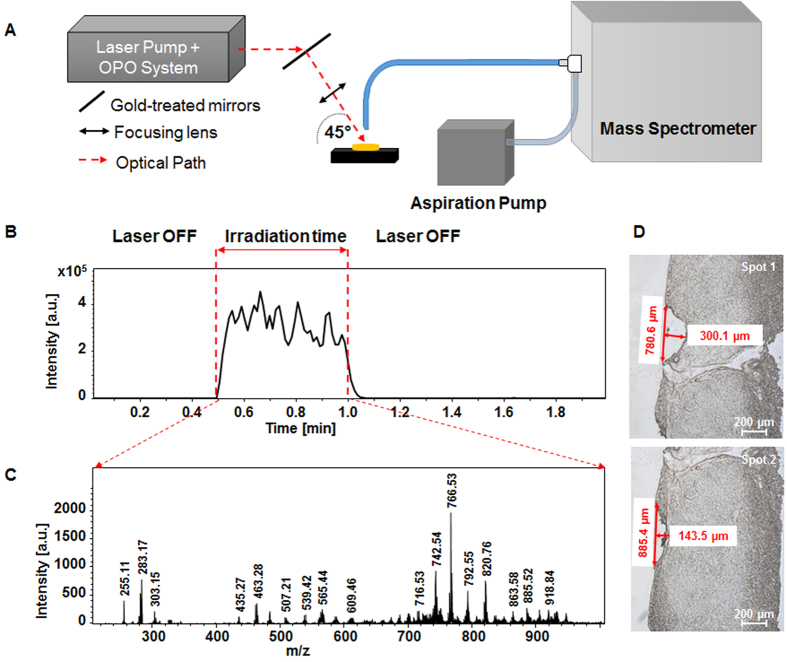
**(A)** Schematic representation of the SpiderMass instrument. **(B)** Typical time evolution of the Total Ion Current (TIC) recorded by the MS instrument for an irradiation period of 30 s and **(C)** Typical MS spectrum in the negative mode extracted by averaging the MS spectra over all the irradiation period. **(D)** Optical microscope images from tissues sections obtained after irradiation with the system of a raw piece of Bovine liver allowing to determine the maximum ablation depth.

The SpiderMass instrument is divided in three different parts, namely 1) a micro-sampling probe, 2) a transfer line and 3) the MS analyzer. Because we aim to develop a system for *in vivo* analysis on humans for clinical applications such as guided surgery or diagnostic purposes, we need the system to be flexible and compatible with introduction into the human body. Sampling was thus designed so that it can be performed remotely from the MS analyzer itself. Because of the requirements of low invasiveness, biocompatibility and remote operation, our micro-sampling probe is based on laser ablation in the IR tuned to excite the most intense vibrational band (O-H stretching mode) of water molecules. This was chosen because all biological tissues contain water and the water content of the human body is high (about 70–80% for most human tissues) making the system operational on all biological tissues. The O-H stretching band of water is lying in the 2.9–3.1 μm range according to the state of water molecules (2.9 μm for liquid water and shifted to 3.1 μm for ice). The instrument was equipped with an optical parametric oscillator (OPO) system pumped by an Nd:YAG laser in order to be able to finely tune the laser wavelength to match the most intense absorption band of water molecules within their biological environment. MS analyzers require gas phase ions. Pure laser ablation only provides low ionization yields especially in the lower fluence regime. Use of matrix molecules absorbing at the laser wavelength was demonstrated to allow for both effective desorption and ionization of molecules and is the basis of the established Matrix Assisted Laser Desorption Ionization (MALDI) ion production sources^30^,^31^,^32^. Here, water molecules, which are present to a large extent in tissues, can then act as an endogenous matrix and provide ionization and prevent the analytes from fragmenting as in conventional MALDI. Few studies reported the use of water as matrix for IR-MALDI. First standard proteins trapped in ice crystals were shown to be detected in 1996 by Berkenkamp and coll.^33^ in conventional vacuum MALDI using a mid-IR laser at 2.94 μm. In 2002, Laiko *et al*. also used water for analyzing standard peptides under IR-AP-MALDI conditions^9^. More recently, Li *et al*.^34^,^35^ demonstrated the possible detection of saccharides metabolites from plant leaves taking advantage of the natural water content of the plant as endogenous matrix. Thus we expect our micro-sampling probe to be both of low invasiveness and provide ions out of intact molecules with sufficient ion yields to achieve good sensitivity MS analysis without requirement of any post-ionization device. The second part of the instrument is the transfer line. The transfer line must retro-transport the micro-sampled material in real-time to the MS instrument. In order to be able to access at least 1–2 m remote measurements between the micro-sampling and the MS instrument, achieve the shortest transfer time, and avoid cross-contamination between real-time experiments, we decided to simply use a PTFE tubing. Indeed tubing systems were previously shown to allow for transporting ions over distances of 1–3 m with preserving sufficient amount of ions for MS analysis^36^,^37^,^38^ and thus appears to be the most efficient and simplest solution to be implemented on the system while avoiding cross-contamination problems. The tubing is positioned just above the material to sample and is directly connected to the inlet of the MS instrument allowing to directly deliver the aerosol. The third part of the setup is a conventional mass spectrometer, here a 3D ion trap mass analyzer. Figure 1 gives a general representation and workflow of the instrument. Figure 1A displays a schematic representation of the SpiderMass instrument. For real-time operation, the MS instrument acquisition is started and signal evolution is monitored in time using the Total Ion Current (TIC) measured at the MS detector for each m/z scan (Fig. 1B) and the corresponding MS spectrum (Fig. 1C). Figure 1B shows the TIC evolution versus time for a typical acquisition on a bovine liver tissue. Until the laser is switched on, only noise level signal is monitored. Immediately when activating the laser, the ion current is observable and remains stable until the laser is turned off, as represented by the dotted lines in Fig. 1B. Each m/z scan acquired by the MS analyzer can be individually extracted out of the TIC to plot the MS spectrum recorded at a definite time of the sequence. Alternatively, if the analyzed surface is homogeneous or if the sample is not moved under the laser beam, an average MS spectrum can be extracted out of a time period. Since the used model tissue (bovine liver) is homogenous, Fig. 1C corresponds to the average MS spectrum extracted out of the whole irradiation period (here 30 s laser irradiation). As observed on the MS spectrum, most of the signals are lying in the m/z [200–1000] range and are to be attributed to lipid species (identification will be discussed later). A video presenting the operation of SpiderMass on the bovine liver tissue can be found in the Supplementary Material (SuppData1), showing a white line trace that corresponds to the irradiation pathway. Because the system is fixed to excite water molecules, local dehydration is induced by laser irradiation leading to this dried white aspect. Other than this effect, no more important damages were observable at the tissue level. The laser beam penetration depth is mainly related to the optical absorption coefficient of water molecules at the used laser wavelength (~3 μm) and is expected to lie within the μm range. Careful examination under a microscope of the tissues after experiments (by cutting thin tissue sections in a plane orthogonal to the analyzed surface) shows a maximum ablation depth of 500 μm for 100 laser shot (30 s irradiation, 10 Hz) (Figure S-1 SuppData 2) although most of depth values lie below 400 μm giving an average ablation depth of 2–5 μm/laser shot (Fig. 1D) in good agreement with the theoretical estimations derived from the absorption coefficient (10^3^–10^4^ cm^−1^ at 3 μm). From these observations the average ablated volume is determined to be in the 0.1–0.3 mm^3^ range for 100 laser shots, thus a recalculated volume of 1–3 10^–3^ mm^3^/laser shot, using here an X-Y spatial resolution of 800 μm × 1000 μm. The spatial resolution can be further decreased to a few hundreds of μm diameter, thus allowing for further decreasing the sampled tissue volume.

### Laser Ablation probe

**Figure 2:**
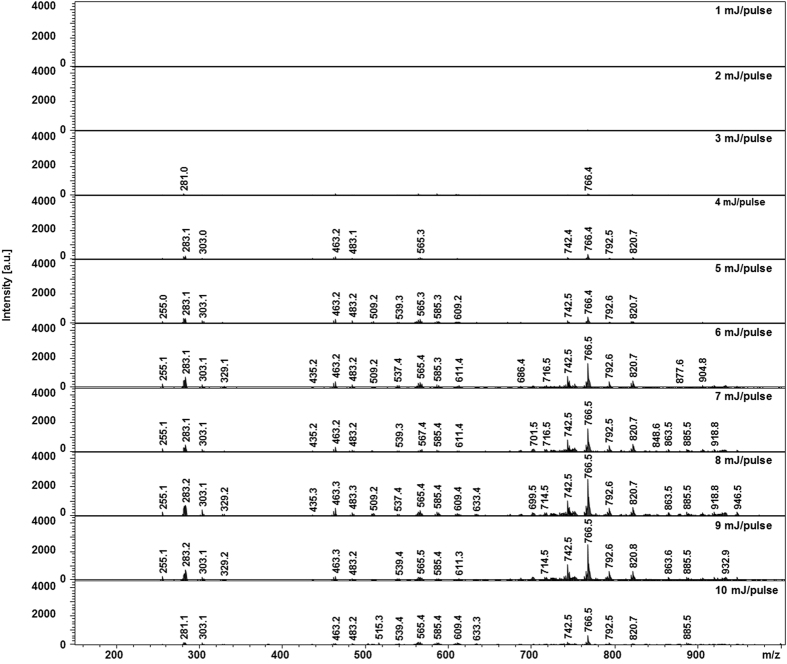
Evolution of the MS spectrum averaged over the whole irradiation period (30 s) in the negative mode with the laser energy. Experiments were performed on a raw piece of bovine liver.

**Figure 3:**
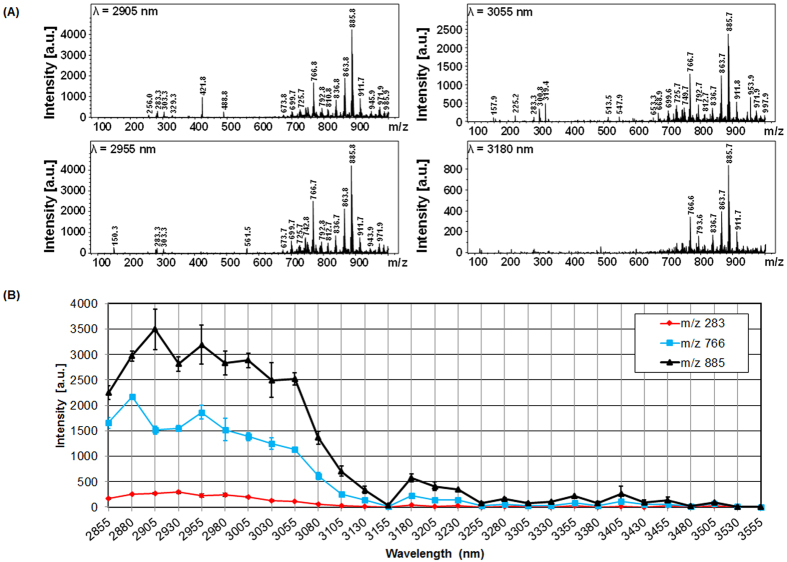
Evolution **(A)** of the MS spectrum averaged over the whole irradiation period (30 s) in the negative mode with the laser wavelength and **(B)** of the intensity of 3 signals of the MS spectra (m/z 283, 766, 885 nominal values). Experiments were performed on a raw piece of bovine liver.

In order to optimize the operation of the system we first studied the influence of laser energy. The laser energy examined has a range of 1 to 10 mJ/pulse and we performed real-time acquisition in both negative and positive ion modes over 30 s irradiation periods. In order to avoid large damage to the tissues we purposely chose to limit the laser energy to 10 mJ/pulse even though higher laser energy values can be achieved if needed. TIC evolution versus laser energy/pulse in negative mode is presented in Figure S-2 (SuppData2) and corresponding extracted spectra (average over the 30 s irradiation) for two experimental replicates is shown in Fig. 2. As observed from the TIC, significant ion signal is detected for a threshold laser energy of 4 mJ/pulse. Although peaks are observed in the corresponding MS spectra below 4 mJ/pulse the signal intensity remains very low leading to poor S/N ratio. A clear increase is then observed at 6 mJ/pulse. Above this value, the signal intensity reaches a plateau up to 9 mJ/pulse and the number of observed signals remains constant although slightly better reproducibility is obtained for 9 mJ/pulse compared to lower values. At 10 mJ/pulse the signal decreases. This is clearly observed in Figure S-3 SuppData2 by plotting the peak intensity extracted from the MS spectra versus laser energy/pulse for three different ions (m/z 283.1, 463.2 and 766.4). In the positive mode, significant signal is recorded at a lower laser energy threshold of 2 mJ/pulse from the TIC (Figure S-4 SuppData2) but a similar increase as in negative mode up to 8 mJ/pulse is then observed. Importantly, it must be noticed that despite TIC above 1.10^5^ (a.u.) the extracted MS spectra show a much lower number of signals and of lower intensity in positive mode (Figure S-5 SuppData2) compared to negative mode. This important difference in negative versus positive mode over the m/z [100–1000] range is explained by the fact that phospholipids are naturally carrying a negative charge on their phosphate groups and can thus be more easily detected in the negative ion mode. Conversely, more small molecules or atomic contaminants carrying a positive charges can contribute to explain important TIC signal while not being associated with useful signal for the application. Thus, we preferentially chose to use negative ion mode at a laser energy between 7–9 mJ/pulse for further experiments. Interestingly, ions are observed reproducibly with ample sensitivity in real-time remotely performed without requiring an ion source in front of the MS spectrometer (conventional ESI) or post-ionization for ionization neutrals. We then further studied the influence of the irradiation wavelength by finely tuning the OPO in the 2.855–3.555 μm range. Evolution of the MS spectra from a total period of 30 s irradiation at 7 mJ/pulse is presented in Fig. 3. Clearly the highest number of signals and the highest signal intensity were obtained for values lying between 2.9 and 3.0 μm. Below 2.9 μm and above 3.0 μm the signal is gradually decreasing over the explored range. This clearly demonstrates a resonant effect with the O-H stretching band, *a priori*, of water molecules at least. IR-MALDI using water as matrix was previously report^9^,^34^. Since in these experiments we observe a similar trend of evolution for the signals of the different molecules detected and the used fluences (about 0.8–1.5 J/cm^2^) are lying in the range of used fluences in IR-MALDI, this encourages the hypothesis of an IR-MALDI-like mechanism for ion formation with water molecules acting as endogenous matrix. Tissue endogenous water molecules present all the characteristics of a MALDI matrix namely absorbing at the laser wavelength and present in large excess compared to analytes.

### Ion transfer line

**Figure 4:**
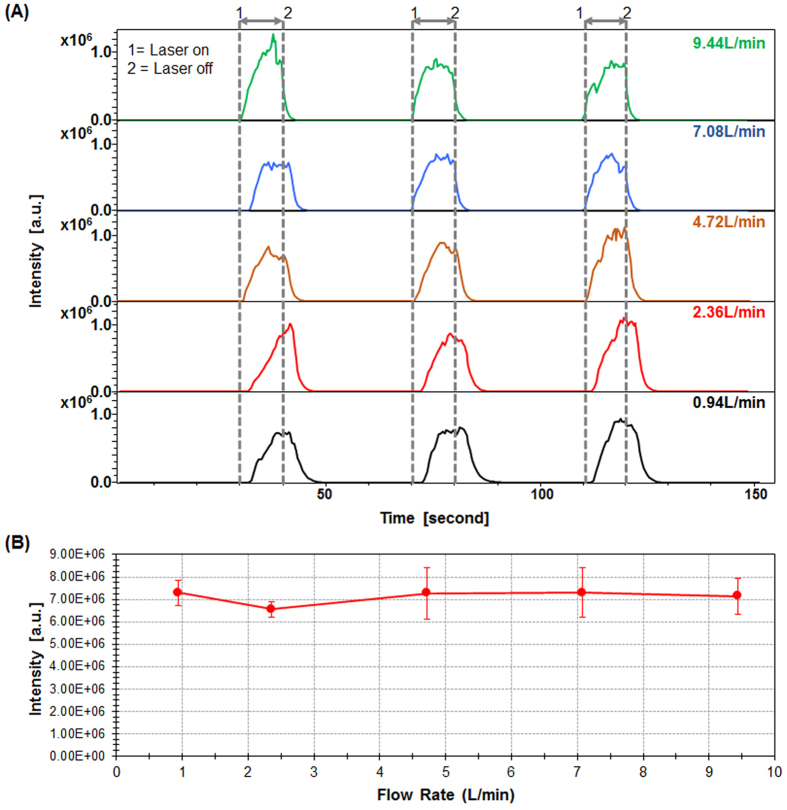
Evolution of the TIC with time for different aspiration flowrates applied on the transfer tube. **(A)** Real-time profiles of TIC evolution along acquisition time. The acquisition includes 3 periods of irradiation of 10 s each. “1” indicates the time at which the laser is switched on and “2” the end of the irradiation period. **(B)** Averaged TIC intensity plotted versus aspiration flowrates. Experiments were performed on a raw piece of bovine liver.

**Figure 5:**
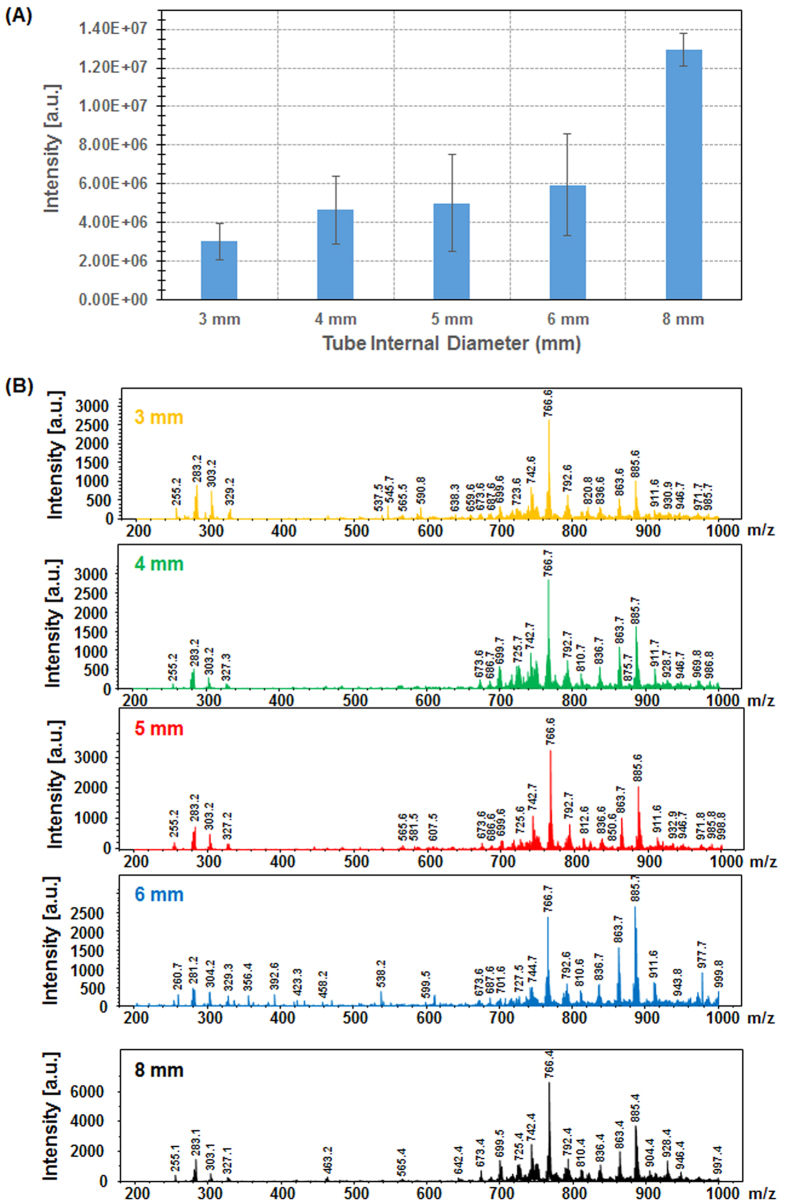
Evolution of **(A)** the TIC in time and **(B)** the MS spectrum averaged over the whole irradiation period (10 s) for different transfer tube diameters. Experiments were performed from a raw piece of bovine liver.

For clinical applications such as guided surgery the transfer line must fulfill two important requirements namely i) compatible with introduction into human body via an endoscopic system if required, which means small tubing diameters, and ii) allows for as short as possible transfer time. The SpiderMass transfer line is connected to the MS inlet as well as a pumping system to control the aspiration by variation of the pressure inside the transfer tube. Figure 4 shows the evolution of TIC with the aspiration flow rate of the vacuum pump for three consecutive laser irradiation periods of 10 s each at a laser energy of 9 mJ/pulse. At low flow rate there is a time-lapse between the start of laser irradiation and the detection of ions by the MS instrument as well as a delay for signal disappearance after deactivating the laser (Fig. 4A). From 0.94 L/min up to 9.44 L/min, the time-lapse for signal observation is decreased by a factor 3 (1.5 ± 0.7 seconds to 0.4 ± 0.1 seconds respectively) when the signal disappearance delay is decreased by a factor 2 (7.9 ± 1 seconds to 3.5 ± 0.3 seconds; respectively). Interestingly, changing the flowrate does not affect the TIC (Fig. 4B) nor the MS spectra even though at the highest flowrate a low negative pressure is created versus the MS inlet. The stability of the MS molecular profiles with the aspiration flowrate is another argument in favor of early ionization of molecules. Indeed, further ionization of molecules within the transfer line should be affected by the aspiration flowrate of the system. Transfer tubes of diameters between 10 down to 3 mm were tested on the system. A decrease in total ion intensity was observed when decreasing the transfer line diameter (Fig. 5A) but most importantly for applicability aspects the molecular profiles were not affected (Fig. 5B) with S/N ratios far good enough for data processing. Tubing with at least down to 3 mm external diameter can be used which makes the system usable for the foreseen applications.

### Identification of detected molecules

**Figure 6:**
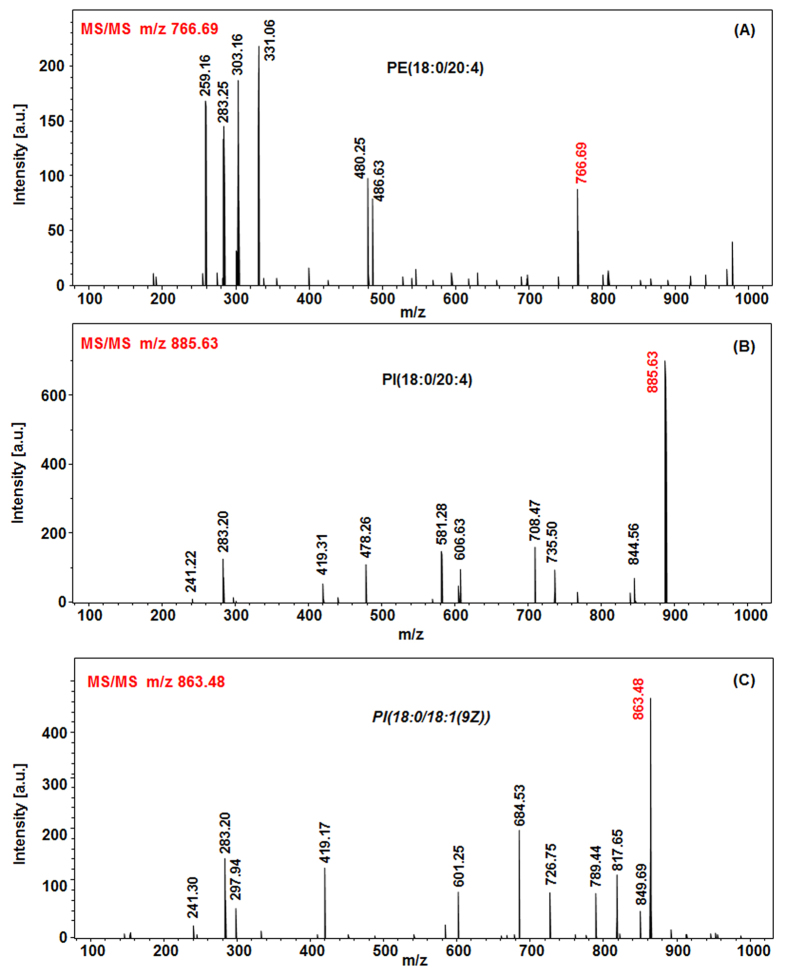
Real-time MS^2^ spectra of three signals observed in the MS spectrum averaged over the whole irradiation period and structure of the identified lipids **(A)** MS^2^ of m/z 766.69 (PE 18:0/20:4), **(B)** MS^2^ of m/z 885.63 (PI 18:0/20:4) and **(C)** MS^2^ of m/z 863.48 (PI 18:0/18:1).

Most of signals observed using the SpiderMass system regardless of the ablation parameters used are lying within the m/z 200–1000 range. Based on the m/z values and profiles they most probably correspond to various lipids constituting cells. We confirm their nature by performing MS^2^ experiments under real-time conditions. Figure 6 shows typical MS^2^ spectra recorded under low collision energy ion activation of molecules showing the highest occurrence in these analyses. Precursor ion m/z 766.69 (Fig. 6A) is attributed to phosphatidylethanolamine PE (18:0/20:4). The typical head group fragment (m/z 140.01) of PE is not observed because of the low mass cut off of the 3D IT instrument. m/z 283.25 and 303.16 can be attributed respectively to Sn1 and Sn2 chains and the m/z 480.25 corresponds to the loss of the Sn1 chain (loss of CH_3_-(CH_2_)_15_-CH-C=O). MS^2^ spectrum in Fig. 6B of precursor ion m/z 885.63 shows the typical fragment ion of a Phosphatidylinositol head group (m/z 241.22). Other fragments typical from the Sn1 chain (m/z 283.2) or the loss of chain Sn2 (m/z 581.28) allow to identify this lipid as phosphatidylinositol PI (18:0/20:4). Finally, parent ion m/z 863.48 MS^2^ spectrum (Fig. 6C) allows to assign this species as phosphatidylinositol PI (18:0/18:1). Interestingly the ion current is stable enough along the irradiation time, thus automatic MS^2^ spectra is possible, monitoring a predefined m/z list of compounds of interest. Using real-time MS experiments we can confirm that observed signals correspond to different families of lipids including phospholipids of various classes (PA, PE and PI) as well as fatty acids (FA) or diacylglycerols (DG). A table summarizing all the identified lipids from raw pieces of bovine liver tissues is presented Table S-1 SuppData2. This identification was confirmed by extraction of lipids using Folch method from the analyzed tissues and further ESI MS & MS/MS analysis on a HR MS instrument (Table S-2 SuppData2). Interestingly, the lipids are detected in their intact form as negative [M-H]^−^ ions in most cases. No fragmentation was shown as proven by the fact that no increase of the signal corresponding to FA species was observed concomitantly to the decrease of the molecular ion of phospholipids when increasing laser energy. This is also confirmed by studies on standard phospholipids. The ability to preserve intact species throughout the experiments demonstrates that ions carry a low enough internal energy so they are stable over the time period of the experiment and reinforces our conviction that ions are created form the very beginning of the ablation according to a MALDI-like mechanism with water as matrix.

### *Ex-vivo* real-time application of the SpiderMass system to cancer

**Figure 7:**
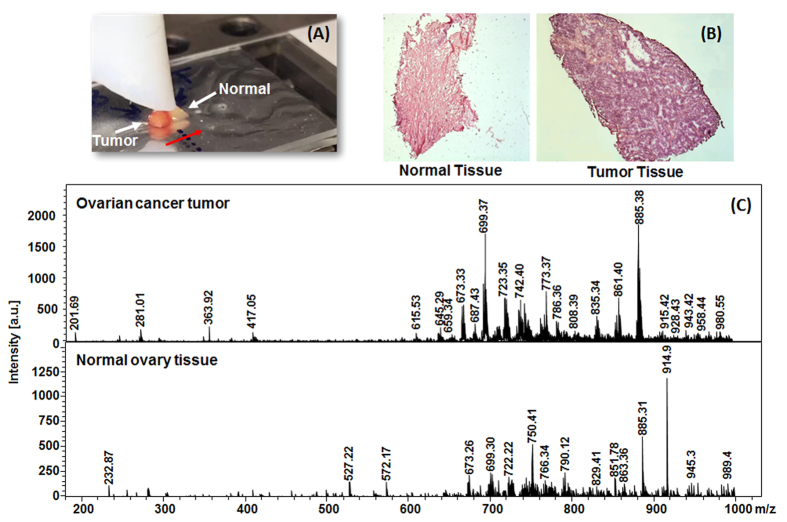
Real-time acquisition on two biopsies of a single patient with ovarian cancer (one biopsy corresponds to the tumor region and the second one is a normal tissue from the bilateral control taken in the second ovary). The red arrow indicates the laser beam axis of translation over the biopsies. **(A)** Photography of the biopsies on the sample stage of the instrument just before starting the experiment. **(B)** HES staining of a tissue section cut out from the analyzed biopsies after the experiment and **(C)** MS spectrum averaged from the whole irradiation period of each biopsy.

Since we could demonstrate that our system is able to retrieve molecular information from intact lipid species in real-time from raw pieces of tissues, we then turn to determine the ability of our system for clinical applications. Figure 7 presents the MS spectra recorded in real-time from two raw tissue pieces of distinct biopsies of ovarian cancer using a laser energy of 5 mJ/pulse. The two biopsies were obtained from the same patient, one from the primary tumor site and was diagnosed to be high grade serous ovarian carcinoma by the pathologist, and the second is a piece of normal tissue taken from the second ovary (bilateral control). The MS spectra are averaged over 30 s while slowly moving the tissue under the laser beam. Clearly these spectra show different profiles. Certain abundant lipid species such as m/z 885.4 (PI 18:0/20:4) are observed from both normal and tumor tissue. On the contrary many signals are specific either to tumor or normal ovary tissue or show a different intensity. For example, m/z 750.40 or 914.97 are only observed for the analysis of normal ovary while m/z 861.40 and 835.34 are specific to ovarian tumor. Thus the molecular profiles can be recorded with low damages to the biopsy tissues and be used to differentiate cells of different phenotype. This indicates the potential of the SpiderMass technology to be used for diagnosis purposes.

### *In vivo* applications of the SpiderMass to human skin

**Figure 8:**
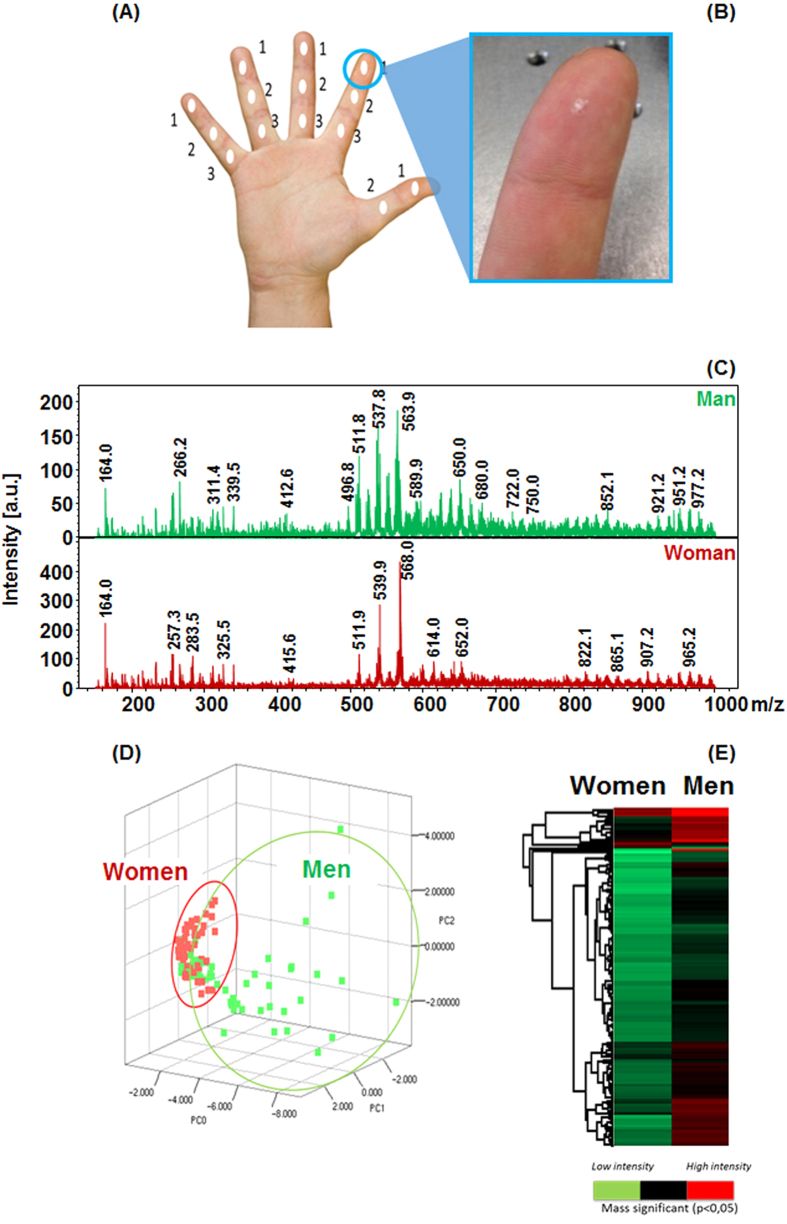
Real-time *in-vivo* analysis using the SpiderMass instrument on human finger skin. **(A)** Schematic mapping of the various irradiation spots on the different phalanxes. **(B)** Picture of a finger after irradiation. The white dehydrated spot marks the irradiation zone. **(C)** Averaged MS spectrum recorded from the same phalanx of a man versus a woman. **(D)** Principal component analysis (PCA) performed from the MS spectra of all the volunteers and showing the discrimination according to gender. **(E)** Hierarchical Clustering (HC) performed from the significant masses (p < 0.05) according the gender.

**Figure 9:**
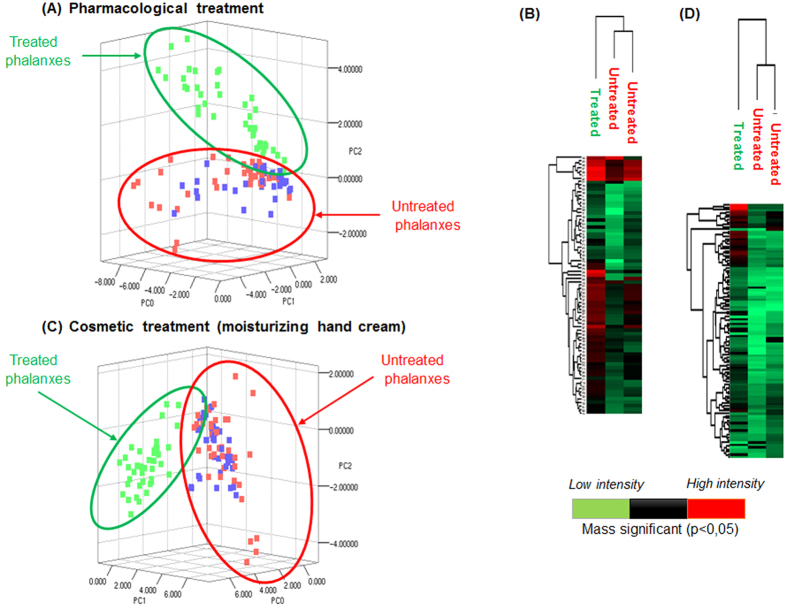
Statistical analyses performed from the MS data recorded on the human phalanxes after treatment with a drug (antalgic) or a cosmetic (moisturizing hand cream). PCA **(A**–**C)** and HC from the significant masses (p < 0.05) **(B**–**D)** performed from the data recorded on the phalanxes treated with the antalgic **(A,B)** or hand cream **(C,D)** versus the untreated phalanxes.

We know from *ex-vivo* experiments performed on tissue pieces that the damages induced by the laser ablation at the wavelength and energy used are low. The performances of the system for real-time *in-vivo* analysis were studied by analyzing in real-time fingers of humans. In these experiments, each phalanx of the right hand (Fig. 8A) is sampled with the system from a cohort of 10 volunteers. Figure 8B shows the aspect of the finger skin after 10 s irradiation. As for biopsies or raw pieces of tissues a white trace is observable corresponding to dehydration. This trace disappears after a few minutes when the skin rehydrates naturally or faster by wetting hands with water. With respect to pain, volunteers confirmed after the experiments to feel a tingling sensation during irradiation but no pain. After checking the painful and invasive aspect of the system, it was used for the analysis of finger surfaces. Figure 8C shows typical MS spectra recorded in real-time by 10 s irradiation of the human phalanxes showing various signals corresponding to different lipid classes. Notable differences in the lipid molecular profiles of men and women were observed. Indeed, m/z 537.8 and m/z 563.9 are for example characteristic of men’s profiles, while m/z 539.9 and m/z 568.0 are related to women. Statistical analysis using Principal Component Analysis (PCA) (Fig. 8D) and hierarchical clustering (HC) (Fig. 8E) permits to discriminate the volunteers according to gender by their MS profiles and retrieve significantly variant m/z according to gender. To check the potential of the SpiderMass for dermatologic applications we further investigated fingers treated with either moisture hand cream or analgesic cream (formulated commercial product). Treatments were applied on one phalanx and comparison of the treated (phalanx 2) and non-treated phalanxes (phalanxes 1 and 3) were performed for all of the individuals after checking that the phalanxes present a similar molecular profile before treatment. Just a few minutes after treatment, the finger was analyzed by performing irradiation along a line going through phalanxes 1 to 3 by displacing the finger under the laser beam. Phalanxes 1 and 3 present a similar profile in contrast to phalanx 2 and show both MS peaks and specific signals in common to Phalanx 1 and 3 (Figure S-6 SuppData2). These specific signals result either from the product itself or a modification of lipid content of the skin (sebum and *stratum corneum*) in reaction to the product application. Statistical analysis using PCA (Fig. 9A) on the data collected for each phalanx (1–3) of the ten volunteers reveals that phalanxes 1 and 3 are classified together and phalanx 2 apart. HC (Fig. 9B) obtained from the m/z with statistical significance (p-value < 0.05) shows a clear separation between untreated and treated skin. A similar experiment was performed using a moisturizing hand cream. Again the treated phalanxes showed a different molecular profile and statistical analysis performed on the cohort of tested individuals demonstrated that the treated skin has a different classification than the untreated one (Fig. 9C,D). Importantly the molecular profiles recorded for the analgesic and moisturizing hand cream are different to the one of the untreated phalanx and are highly different from each other (Figure S-7 SuppData2).

The experiments demonstrate the possible use for *in-vivo* real-time analysis of the system with minor damages to the tissues and painless. It also demonstrate the potential of the system for applications such as dermatology or cosmetology.

## Discussion

The present study demonstrates the possible development of a MS-based system allowing for *in vivo* real-time analysis of biological tissues with low damages to the tissue and painless. The SpiderMass system is based on *in-situ* gas phase ion production under laser ablation by excitation of the O-H stretching band of the water molecules present in biological tissues using IR laser. We demonstrate that these ablation conditions are compatible with *in vivo* applications and allow to generate sufficient gas phase ions that can be directly transferred to the mass analyzer without using other means of ion production (ion source or post-ionization) as previously reported (eg. remote LAESI^29^). Thus, using a simple aspirating transfer line, ions can be transferred in the millisecond timescale to the MS instrument allowing for real-time analysis. The main parameters affecting ion production are the laser wavelength and the laser energy. We can show a real resonance effect with the O-H stretching mode of water molecules since most intense signals are generated between 2.9 and 2.95 μm. With respect to the number and intensity of signals, optimal laser energy lies in the range between 5 to 9 mJ/pulse. In general, the relatively low laser fluence used (about 0.8–1.5 J/cm^2^), the observation of ions corresponding to intact species of molecules, the similar behavior of the different biomolecules beside their different physico-chemical properties, the absence of fragmentation, and the low influence of the aspiration flowrate in the transfer line on the molecular profiles suggest that ions are produced according to an IR-MALDI-like mechanism. Many applications can be foreseen for the SpiderMass system. *Ex-vivo* analysis of raw tissue pieces of patient biopsies demonstrate that specific molecular profiles are obtained from normal or cancerous tissues. We also demonstrate the low invasiveness of the analysis and the possible use of the small transfer tubing for further miniaturization of the system as well as the real-time capabilities. One of the major applications foreseen for the system is to be used as a tool for guided surgery of cancers. By scanning the patient tissues with the laser, surgeons can retrieve molecular profiles in real-time and obtain a diagnostic by comparison to a molecular databank built for a given pathology in a similar operation as the iKnife system^24^,^26^,^39^. However, unlike the iKnife system, SpiderMass has the major advantage of being less invasive since only an estimated volume of 0.1–0.3 mm^3^ is sampled. Application to the screening of human fingers skin confirm the low invasiveness of SpiderMass and demonstrate possible application within the field of dermatology and cosmetology. Although the system has been designed for operating in the surgery room for guided surgery allowing the surgeon to define the tumor margins, find secondary low volume tumor sites and allow to retrieve tumor stage, SpiderMass can easily be adapted to many other applications. Basically the system is operating on all biological tissues (and any system containing water) and applications within the field of food quality control and safety (identification of pesticides from food, checking meat quality…) or environmental issues can be envisaged. Figure S-8 (SuppData 2) presents the real-time analysis from a raw piece of an apple. This apple piece shows a normal part and a rotten part. SpiderMass was used to analyze both the outside of the apple piece (apple skin) and the inner part (apple core). We observe that the molecular patterns observed from the apple skin and core are largely different. Both specific m/z ratio are observed as well as relative intensity differences. Similar observations are made for the analysis of the normal and rotten part both for the apple skin and the core part. This gives a possible example of applications of the system for agricultural and food safety considerations.

In conclusion, we were able to design and setup a new instrument based on mass spectrometry for *in-vivo* real time analysis allowing to retrieve molecular information which permits for *in-situ* ion production and direct transfer to the mass analyzer from any raw piece of biological tissue. In the future we will look for miniaturizing the system and facilitate its ease of use by implementing laser beam delivery through fiber optics. This will give more degrees of freedom to the system by allowing the user to scan a surface with the laser, and by allowing its introduction into a small volume by a small aperture as might be requested for application to surgical conditions. Finally, by simply tuning the laser wavelength to other bands than water vibration modes, applications to surface analyses for organic molecule constituents such as in rocks or other surfaces might also be envisaged using the SpiderMass.

## Methods

### Tissues for *ex-vivo* analysis

All of the optimizations were performed using pieces of bovine liver purchased from a local market. The ovarian biopsies were obtained from patients of the Centre Oscar Lambret (Lille, France) and from the CHRU de Lille Pathology Department. All experiments were approved by the local Ethics Committee (CPP Nord Ouest IV 12/10) in accordance with the French and European legislation on this topic. Methods of collection for human ovaries were performed in accordance with procedures that were approved by the Ethics Committee of the CHRU Lille.

### Human volunteers

A cohort of ten healthy volunteers including 5 men and 5 women was selected for the experiments without any discrimination according to age. Before the experiments they signed an informed consent authorization form describing the experimental protocol and instrument and exposure to the hazards. No personal information such as the name of the individuals were used in these experiments. A randomized number was assigned to each individual.

### SpiderMass instrument setup

A homemade instrument based on laser irradiation and real-time analysis of ejected materials by mass spectrometry (MS) was developed for the experiments. An infrared OPO (LaserSpec, Malonne, Belgium) pumped by a 4 ns Nd:YAG Quantel Brilliant Eazy laser operating at 1064 nm with a repetition rate of 10 Hz was used for laser irradiation. The OPO beam can be tuned to reach the desired wavelength. A set of reflecting mirrors and a CaF_2_ convergent lens (f′ = 5 cm) were setup on the laser beam pathway to bring the beam onto the sample surface with an angle of 45°. The resulting laser spot is an ellipse of 0.8 mm by 1 mm corresponding to an irradiated surface of 0.628 mm^2^. A laser power meter (Molectron Coherent Corporate, Santa Clara, CA, USA) was used to measure the laser energy per pulse at the exit of the laser. The laser energy per pulse is ranged between 1 and 10 mJ/pulse by using 30 seconds laser irradiation of the same area of tissue pieces of bovine liver (≈0.25 cm^2^). Due to the humidity of air the laser beam loses energy on the pathway to the sample. From measurement taking into account the length of the laser pathway on our setup, we estimate that laser energy is divided by about 3 on the sample. Transfer of ejected particles is insured by a 2 m PTFE tubing. Tubing of 0.3–1 cm inner diameter were tested. The PTFE tubing is connected to the inlet of a 3 D Ion Trap mass spectrometer (HCT Ultra, Bruker Daltonics, Bremen, Germany) with the ion source removed. An aspiration pump (Ted Pella Inc., Redding, CA, USA), equipped with both flowrate and pressure meter, was attached to the PFTE tubing. The connection between the PFTE tubing and the inlet of the mass spectrometer was established by a copper tubing (1.5 mm ID × 3 mm OD; length = 15 cm) and an extension of the transfer capillary used in the case of nanoESI-MS. The copper tubing was inserted into the PFTE tubing. The MS instrument was set to scan the 150–1000 m/z range. The maximum accumulation time in the ion trap was set to 450 ms for 200,000 ions.

### Biopsy Analysis

Two biopsy tissues coming from the same patient and stored at −80 °C were placed in the fridge for slow thawing. Thawed tissues were then placed one beside the other on a glass slide which was set at the adequate position for laser irradiation. MS acquisition in the negative mode was launched from the acquisition panel of the instrument. The laser was then switched on and the slide was slowly moved under the laser beam for 30 s. The experiments were performed at a laser energy of 5 mJ/pulse and a flowrate of 4.7 L/min. The laser was then switched off and the operation was repeated on the second biopsy. After examining the two biopsies, the MS acquisition was stopped and MS spectra were extracted from the TIC. The irradiation periods are easily observed from the TIC and MS spectra were averaged over the 30 s irradiations. Spectra were visualized using the data processing software of the HCT instrument, DataAnalysis v.4 (Bruker Daltonics, Bremen, Germany).

### *In-vivo* Skin Analysis

Three distinct experiments were performed according the same sequence for each individual. The volunteers were asked to place their finger at the adequate position under the laser beam. The MS acquisition was started. The laser was then switched on. The volunteers were asked to slowly move the fingers under the laser beam for a 30 s irradiation period. The laser was then switched off. The MS spectra were averaged over the 30 s irradiation period and extracted from the TIC. For the first experiment the phalanxes of the right hand were irradiated. In the second experiment, on the same hand, a pharmacological product (Anbesol GEL, Whitehall – Robbins INC., Mississauga, Ontario, Canada) was spread onto the second phalanx for each finger except the thumb. At the end of these experiments, volunteers washed their hands. Then after 30 min waiting, a hand cream for dry skin (cosmetic product, L’Occitane, Manosque, France) was spread onto the second phalanx of the fingers and submitted to analysis. After analysis, for each phalanx, peak lists (m/z, intensity) were generated using out of the spectra DataAnalysis v.4 (Bruker Daltonics, Bremen, Germany) between the mass m/z 150–1000 with signal-to-noise ratios higher than 3 for each detected ion. The peak lists were then exported to Mass-Up software (SING Group, Ourense, Spain). Intra-sample and inter-sample peaks matching were performed on peak lists to realize supervised principal component analysis (PCA). For hierarchical clustering, spectra were aligned and peak lists were exported to the Perseus software (www.perseus-framework.org; v. 1.5.0.31). The annotated matrices were first transformed using log2 (intensity) and then an appropriate sample test was performed. To obtain an interpretable cluster, the expression data with the same categorical annotation were averaged over group forms. Finally, the data were filtered to keep only the significant m/z according to the previous sample test (p-value < 0.05).

### Real-time analysis of apple

A thick section (few mm) was taken out of an apple presenting a rotten part using a scalpel. The resulting piece of tissue (Figure S-7 SuppData 2) was scanned in real time along a line crossing the entire section from top to bottom and passing by both the normal and rotten regions. The real-time analysis was performed both from the inside and the outside (apple skin) of the fruit at a laser energy of 5 mJ/pulse and a flow rate of 4.7 L/min.

## Additional Information

**How to cite this article**: Fatou, B. *et al*. *In vivo* Real-Time Mass Spectrometry for Guided Surgery Application. *Sci. Rep*. **6**, 25919; doi: 10.1038/srep25919 (2016).

## Supplementary Material

Supplementary Information

Supplementary Movie S1
