# Supplementary material for: In vivo Real-Time Mass Spectrometry for Guided Surgery Application
Source: Sci Rep. 2016 May 18;6:25919. doi: 10.1038/srep25919 (PMC4870577; doi:10.1038/srep25919)
Supplement: Supplementary Information [file srep25919-s1.doc]

**I*n vivo* Real-Time Mass Spectrometry for Guided Surgery Application**

**Benoit Fatou^1,2^, Philippe Saudemont^1^, Eric Leblanc^1,3^, Denis Vinatier^1,4^, Violette Mesdag^1,4^, Maxence Wisztorski^1^, Cristian Focsa^2^, Michel Salzet^1^, Michael Ziskind^2*^, Isabelle Fournier^1*^**

^1^ Univ. Lille, INSERM, U1192 - Laboratoire Protéomique, Réponse Inflammatoire et Spectrométrie de Masse-PRISM, F-59000 Lille, France

^2^ Univ. Lille, CNRS, UMR 8523 - PhLAM - Physique des Lasers Atomes et Molécules, F-59000 Lille, France

^3^Department of Gynecology Oncology, Cancer Center Oscar Lambret, Lille, France

^4^ Département Universitaire de Gynécologie Obstétrique, Service de chirurgie gynécologique. Hôpital Jeanne de Flandre, CHRU de Lille, 59037 Lille Cedex

**Corresponding Authors:**

**Prof. Isabelle Fournier.** E-mail: [isabelle.fournier@univ-lille1.fr](mailto:isabelle.fournier@univ-lille1.fr) Tel: +33 (0)3 20 43 41 94, Fax: +33 (0)3 20 43 40 54.

**Dr. Michael Ziskind.** E-mail: [michael.ziskind@univ-lille1.fr](mailto:michael.ziskind@univ-lille1.fr) Tel: +33 (0)3 20 33 63 30, Fax: +33 (0)3 20 33 64 63.

**Supp Data 1:** Video showing the operation of the SpiderMass system in real-time on a raw piece of tissue (bovine liver). Experiments were performed from a raw piece of bovine liver.

[SupplementaryData1_video_IsabelleFournier.MOV](../../Revision_Publication_Benoit_SpiderMass_SciReport_Submission/SupplementaryData1_video_IsabelleFournier.MOV)

**
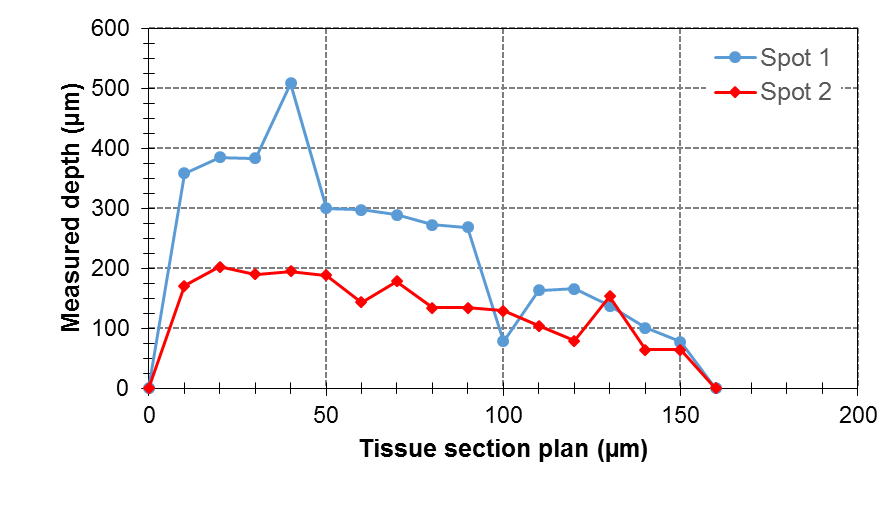
**

**Supp Data 2; Figure S-1:** Evolution of the irradiation spot depth measured under microscope after a 10s irradiation (100 laser shots, 10 Hz) without moving the laser beam at 7mJ/pulse on a bovine liver raw piece of tissue on two distinct irradiation spots. After irradiation, tissue was sectioned on a cryostat perpendicularly to the surface of the tissue. 10 µm tissue sections were sliced consecutively and, for each section, irradiation spot depth was measured under a microscope and depth was plotted as a function of the total tissue thickness.

**Supp Data 2; Figure S-2:** Negative mode TIC time evolution for different laser pulse energies. Experiments were performed from a raw piece of bovine liver.

**Supp Data 2; Figure S-3:** Evolution of the intensity of the three most intense signals observed in the averaged MS spectrum over the irradiation period with respect to the pulse laser energy.

**Supp Data 2; Figure S-4:** Evolution of the TIC with time in the negative mode for different laser pulse energy. Experiments were performed from a raw piece of bovine liver.

**Supp Data 2; Figure S-5:** Evolution of the positive mode MS spectrum averaged over the whole irradiation period (30 s) with the laser energy/pulse. Experiments were performed from a raw piece of bovine liver.


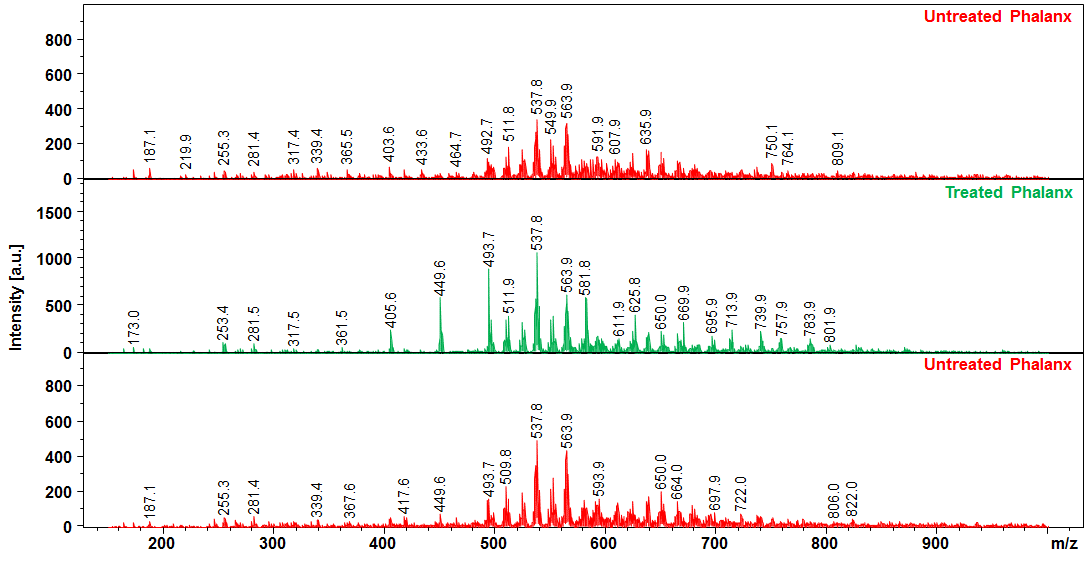


**Supp Data 2; Figure S-6:** MS spectra in the negative mode averaged over in vivo real-time acquisition on human finger skin for treated (analgesic drug application) versus non-treated phalanxes.


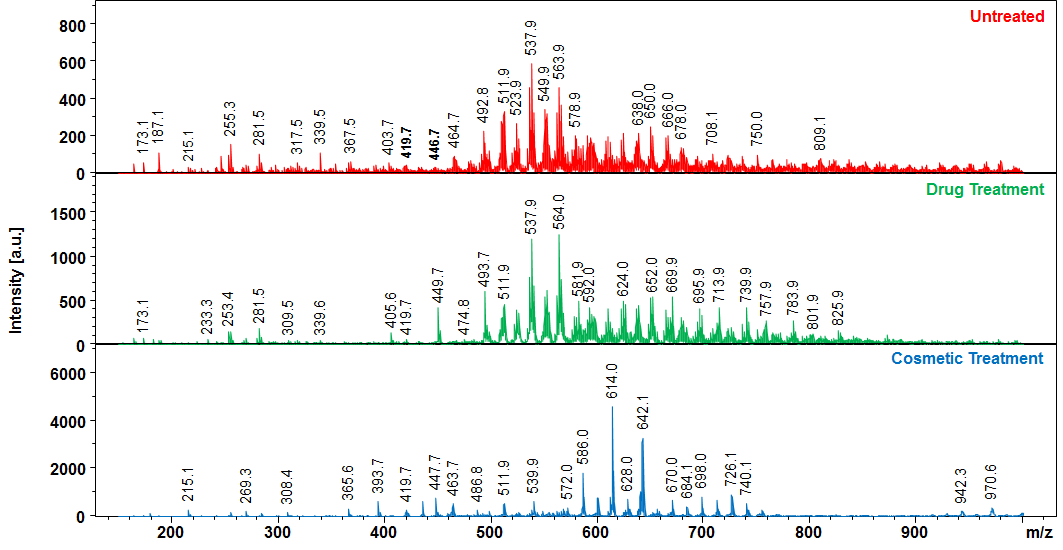


**Supp Data 2; Figure S-7:** MS spectra in the negative mode averaged over in-vivo real-time acquisition on human finger skin for treated with analgesic drug and moisturizing hand cream versus non-treated phalanxes.


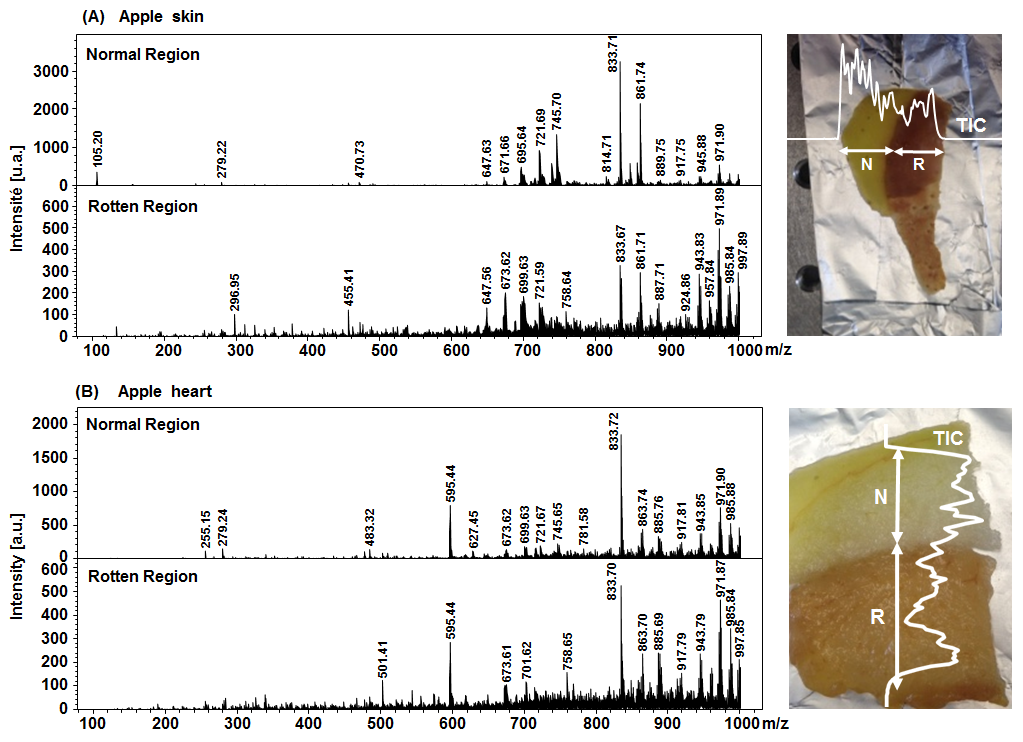


**Supp Data 2; Figure S-8:** Real-time analysis of a raw tissue piece of apple. TIC evolution with time and position and corresponding extracted MS spectra in the negative mode averaged over the time period corresponding either to the irradiation of the normal tissue (N) or the irradiation of the rotten part (R) for both the outer part (apple skin) **(A)** and the inner part **(B)**. During acquisition the tissue is moved at constant speed under the laser beam. TIC timescale is sized to the spatial displacement.

| **Experimental m/z** | **Theoretical m/z** | **Identification** |
| --- | --- | --- |
| 255.11 | 255.23 | Palmitic acid C16:0 |
| 281.16 | 281.25 | Octadecenoic acid C18:1 |
| 283.17 | 283.26 | Stearic acid C18:0 |
| 303.15 | 303.23 | Arachidonic acid C20:5 |
| 327.13 | 327.23 | Docosatriynoic acid C22:6 |
| 435.27 | 435.25 | PA (18:0) |
| 461.25 | 461.27 | PA (22:0) |
| 463.28 | 463.28 | PA (20:1) |
| 483.22 | 483.27 | PG (16:0) |
| 507.21 | 507.27 | PG (18:2) |
| 509.25 | 509.29 | PG (18:1) |
| 511.38 | 511.44 | DG (28:0) |
| 537.38 | 537.32 | PG (20:1) |
| 539.42 | 539.47 | DG (30:0) |
| 565.44 | 565.48 | DG (32:1) |
| 585.38 | 585.34 | PI (O-18:0) |
| 609.46 | 609.45 | DG (36:7) |
| 613.47 | 613.48 | DG (36:5) |
| 673.43 | 673.48 | PA (34:1) |
| 701.56 | 701.56 | PE-Cer (37:1) |
| 726.5 | 726.59 | Plasmenyl-PE (36:2) |
| 742.54 | 742.54 | PE (36:2) |
| 750.52 | 750.59 | Plasmenyl-PE (37:5) |
| 766.53 | 766.54 | PE (38:4) |
| 788.53 | 788.52 | PS (36:1) |
| 792.55 | 792.55 | PE (40:5) |
| 806.7 | 806.55 | ST (18:0) |
| 834.56 | 834.58 | PS (40:6) |
| 836.55 | 836.54 | PS (40:5) |
| 862.56 | 862.61 | ST (22:0) |
| 878.63 | 878.60 | ST (h22:0) |
| 885.52 | 885.55 | PI (38:4) |
| 888.55 | 888.62 | ST (24:1) |
| 904.79 | 904.62 | ST (h24:1) |

**Supp Data 2; Table S-1:** Peaks assignation from the real-time analysis of a raw piece of bovine liver using the SpiderMass system (7mJ/pulse laser energy, 10s rradiation).

| **Experimental m/z** | **Theoretical m/z** | **Lipids**  **Identification** | **m/z MS^2^ Fragments** | **MS^2^ Fragment attribution** |
| --- | --- | --- | --- | --- |
| **480.2995** | **480.3095** | **PE(18:0)** | 418.25 | Neutral loss of ethanolamine(C2H6N) and H2O from [M-H]^-^ |
|  |  |  | 283.26 | C18:0 |
|  |  |  | 214.05 | Loss of C18:0 |
|  |  |  | 196.04 | Neutral loss of C18:0 |
|  |  |  | 153.00 | Glycerol-3-phosphate ion with loss of H2O |
|  |  |  | 140.01 | Ethanolamine phosphate ion |
|  |  |  | 78.96 | PO3- ion (from phosphate) |
| **539.4932** | **539.4681** | **DG(30:0)** | 283.26 | C18:0 |
|  |  |  | 255.23 | C16:0 |
|  |  |  | 99.00 | Glycerol |
| **742.5244** | **742.5392** | **PE(36:2)** | 480.31 | Loss of C18:2 |
|  |  |  | 476.28 | Loss of C18:0 |
|  |  |  | 462.30 | Neutral loss of C18:2 |
|  |  |  | 458.27 | Neutral loss of C18:0 |
|  |  |  | 283.26 | C18:0 |
|  |  |  | 279.23 | C18:2 |
|  |  |  | 153.00 | Glycerol-3-phosphate ion with loss of H2O |
|  |  |  | 140.01 | Ethanolamine phosphate ion |
|  |  |  | 96.97 | H2PO4- ion (from phosphate) |
|  |  |  | 78.96 | PO3- ion (from phosphate) |
| **747.5035** | **747.5181** | **PG(34:1)** | 673.48 | Loss of glycerol from precursor ion |
|  |  |  | 509.29 | Loss of C16:0 |
|  |  |  | 483.27 | Loss of C18:0 |
|  |  |  | 465.26 | Neutral loss of C18:0 |
|  |  |  | 281.25 | C18:0 |
|  |  |  | 255.23 | C16:0 |
|  |  |  | 153.00 | Glycerol-3-phosphate ion with loss of H2O |
|  |  |  | 96.97 | H2PO4- ion (from phosphate) |
|  |  |  | 78.96 | PO3- ion (from phosphate) |
| **764.5081** | **764.5236** | **PE(38:5)** | 498.26 | Loss of C18:0 |
|  |  |  | 480.31 | Loss of C20:5 |
|  |  |  | 480.25 | Neutral loss of C18:0 |
|  |  |  | 462.30 | Neutral loss of C20:5 |
|  |  |  | 301.22 | C20:5 |
|  |  |  | 283.26 | C18:0 |
|  |  |  | 153.00 | Glycerol-3-phosphate ion with loss of H2O |
|  |  |  | 140.01 | Ethanolamine phosphate ion |
|  |  |  | 96.97 | H2PO4- ion (from phosphate) |
|  |  |  | 78.96 | PO3- ion (from phosphate) |
| **788.5295** | **788.5447** | **PS(36:1)** | 701.51 | Loss of serine from precursor ion |
|  |  |  | 437.27 | Loss of C18:1 |
|  |  |  | 435.25 | Loss of C18:0 |
|  |  |  | 419.26 | Neutral loss of C18:1 |
|  |  |  | 417.24 | Neutral loss of C18:0 |
|  |  |  | 283.26 | C18:0 |
|  |  |  | 281.25 | C18:1 |
|  |  |  | 153.00 | Glycerol-3-phosphate ion with loss of H2O |
|  |  |  | 96.97 | H2PO4- ion (from phosphate) |
|  |  |  | 78.96 | PO3- ion (from phosphate) |
| **810.5125** | **810.5290** | **PS(38:4)** | 723.50 | Loss of serine from precursor ion |
|  |  |  | 457.24 | Loss of C18:0 and serine from [M-H]^-^ |
|  |  |  | 439.23 | Neutral loss of C18:0 and serine from [M-H]^-^ |
|  |  |  | 437.27 | Loss of C20:4 and serine from [M-H]^-^ |
|  |  |  | 419.26 | Neutral loss of C20:4 and serine from [M-H]^-^ |
|  |  |  | 303.23 | C20:4 |
|  |  |  | 283.26 | C18:0 |
|  |  |  | 153.00 | Glycerol-3-phosphate ion with loss of H2O |
|  |  |  | 96.97 | H2PO4- ion (from phosphate) |
|  |  |  | 78.96 | PO3- ion (from phosphate) |
| **836.5262** | **836.5447** | **PS(40:5)** | 749.51 | Loss of serine from precursor ion |
|  |  |  | 483.25 | Loss of C18:0 and serine from [M-H]^-^ |
|  |  |  | 465.24 | Neutral loss of C18:0 and serine from [M-H]^-^ |
|  |  |  | 437.27 | Loss of C22:5 and serine from [M-H]- |
|  |  |  | 419.26 | Neutral loss of C18:0 and serine from [M-H]^-^ |
|  |  |  | 329.25 | C22:5 |
|  |  |  | 283.26 | C18:0 |
|  |  |  | 153.00 | Glycerol-3-phosphate ion with loss of H2O |
|  |  |  | 96.97 | H2PO4- ion (from phosphate) |
|  |  |  | 78.96 | PO3- ion (from phosphate) |
| **861.5326** | **861.5498** | **PI(36:2)** | 699.50 | Loss of inositol from [M-H]^-^ |
|  |  |  | 599.32 | Loss of C18:2 |
|  |  |  | 581.31 | Neutral loss of C18:2 |
|  |  |  | 577.28 | Neutral loss of C18:0 |
|  |  |  | 437.27 | Loss of C18:2 and inositol from [M-H]^-^ |
|  |  |  | 419.26 | Neutral loss of C18:2 and inositol from [M-H]^-^ |
|  |  |  | 415.23 | Neutral loss of C18:0 and inositol from [M-H]^-^ |
|  |  |  | 283.26 | C18:0 |
|  |  |  | 279.23 | C18:2 |
|  |  |  | 241.01 | Inositol phosphate ion |
|  |  |  | 223.00 | Inositol phosphate ion - H2O |
|  |  |  | 153.00 | Glycerol-3-phosphate ion with loss of H2O |
|  |  |  | 96.97 | H2PO4- ion (from phosphate) |
|  |  |  | 78.96 | PO3- ion (from phosphate) |
| **883.5160** | **883.5342** | **PI(38:5)** | 599.26 | Neutral loss of C18:0 |
|  |  |  | 581.31 | Neutral loss of C20:4 |
|  |  |  | 437.21 | Neutral loss of C18:0 and inositol from [M-H]^-^ |
|  |  |  | 419.26 | Neutral loss of C20:4 and inositol from [M-H]^-^ |
|  |  |  | 301.22 | C20:4 |
|  |  |  | 283.26 | C18:0 |
|  |  |  | 241.01 | Inositol phosphate ion |
|  |  |  | 223.00 | Inositol phosphate ion - H2O |
|  |  |  | 153.00 | Glycerol-3-phosphate ion with loss of H2O |
|  |  |  | 96.97 | H2PO4- ion (from phosphate) |
|  |  |  | 78.96 | PO3- ion (from phosphate) |
| **887.5459** | **887.5655** | **PI(38:3)** | 621.30 | Loss of C18:0 |
|  |  |  | 603.29 | Neutral loss of C18:0 |
|  |  |  | 599.32 | Loss of C20:3 |
|  |  |  | 581.31 | Neutral loss of C20:3 |
|  |  |  | 441.24 | Neutral loss of C18:0 and inositol from [M-H]^-^ |
|  |  |  | 419.26 | Neutral loss of C20:3 and inositol from [M-H]^-^ |
|  |  |  | 315.05 | Glycerophosphoinositol -H2O |
|  |  |  | 305.25 | C20:3 |
|  |  |  | 283.26 | C18:0 |
|  |  |  | 241.01 | Inositol phosphate ion |
|  |  |  | 223.00 | Inositol phosphate ion - H2O |
|  |  |  | 153.00 | Glycerol-3-phosphate ion with loss of H2O |
|  |  |  | 96.97 | H2PO4- ion (from phosphate) |
|  |  |  | 78.96 | PO3- ion (from phosphate) |
| **911.5470** | **911.5655** | **PI(40:5)** | 749.51 | Loss of inositol from [M-H]- |
|  |  |  | 645.30 | Loss of C18:0 |
|  |  |  | 627.29 | Neutral loss of C18:0 |
|  |  |  | 599.32 | Loss of C22:5 |
|  |  |  | 581.31 | Neutral loss of C22:5 |
|  |  |  | 483.25 | Loss of C18:0 and inositol from [M-H]^-^ |
|  |  |  | 437.27 | Loss of C22:5 and inositol from [M-H]^-^ |
|  |  |  | 419.26 | Neutral loss of C22:5 and inositol from [M-H]^-^ |
|  |  |  | 329.25 | C22:5 |
|  |  |  | 283.26 | C18:0 |
|  |  |  | 241.01 | Inositol phosphate ion |
|  |  |  | 153.00 | Glycerol-3-phosphate ion with loss of H2O |
|  |  |  | 96.97 | H2PO4^-^ ion (from phosphate) |
|  |  |  | 78.96 | PO3^-^ ion (from phosphate) |

**Supp Data 2; Table S-2:** Lipids identification performed using ESI-HR MS & MS/MS on a Q-orbitrap instrument by extraction of lipids using Folch method from the irradiated area of the raw piece of bovine liver tissue used for the real-time analysis (7mJ/Pulse laser energy, irradiation 10s). Lipids were identified based on their mass measurement in the MS and their specific fragmentations in MS/MS using HCD activation. These experiments were performed to confirm the identification of lipids performed during real-time experiments. Are only reported in this table the lipids found in common to both experiments.
